# Supplementary material for: Anthropometric and strength characteristics of adolescent golfers with low and high handicaps
Source: PLoS One. 2025 May 20;20(5):e0324065. doi: 10.1371/journal.pone.0324065 (PMC12091782; doi:10.1371/journal.pone.0324065)
Supplement: Attachment — Submitted filename: Authors response Comment.pdf [file pone.0324065.s004.pdf]

Manuscript Number

PONE-D-24-29304

Abstract: Validity and reliability of the test should be mentioned.

Author's reply: Thank you for your valuable suggestion. We agree with the reviewer's suggestions and have incorporated the recommended revisions into the Abstract.We have included detailed validity and reliability information of the testing methods used in the 'Strength and Power Test s'subsection within the Methods section. Specific ICC values and relevant references were added to clearly demonstrate the robustness of our measurement protocols. highlighted in green.

Introduction: The statement of the problem is not mentioned well. What was the need to do this? Explain this problem?

Author's reply: Thank you for highlighting this issue. We have clarified the statement of the problem more explicitly in the revised Introduction section, clearly indicating the gap in existing literature regarding adolescent golfers and the necessity of exploring differences in anthropometric and strength characteristics between skill levels. Specifically, we emphasized that adolescence is a critical developmental period, and identifying these differences is essential for designing effective training interventions, optimizing talent identification, and informing long-term athlete development.

Materials and Methods: 1. validity, reliability and source in the tests of maximum strength, throwing the ball forward, backward, right and left.

Author's reply: Thank you for pointing out this important issue. After carefully reviewing the referenced literature, we found that specific ICC values for the directional medicine ball throws (forward, backward, right, left) were not explicitly reported. Therefore, we have revised our manuscript to accurately reflect that while medicine ball throws are widely accepted and used as valid field tests to assess explosive power, specific ICC values for these directions are not explicitly stated in existing references. This clarification ensures the accuracy and integrity of our description. highlighted in green.

2. In the anthropometric tests of arm circumference, chest and arm width, refer to the source.

Author's reply: Similarly, anthropometric measurements including arm circumference, chest circumference, and shoulder width were performed following standardized ISAK protocols, with relevant references clearly indicated. highlighted in green.

Discussion: 1. In general, in the field of strength test, more comprehensive explanations are necessary to complete this section.

Author's reply:Thank you for highlighting this issue. In response to your suggestion, we have further expanded the 'Strength and Power Tests' subsection by providing more explicit details regarding the testing procedures, including participant positioning, test equipment, number of trials, and specific operational guidelines for each strength and explosive power measure. We

believe this level of detail will ensure clarity, reproducibility, and enhance the scientific rigor of our methods section. highlighted in green.

2. You have to use more tricks to complete this section

Author's reply: Thank you very much for your valuable feedback. In response to your suggestions, we have already spent considerable time and effort significantly revising and enhancing the Discussion section. We believe the current revision has substantially improved clarity, structure, and academic rigor, effectively addressing all your previous comments.
