# Supplementary material for: Anthropometric and strength characteristics of adolescent golfers with low and high handicaps
Source: PLoS One. 2025 May 20;20(5):e0324065. doi: 10.1371/journal.pone.0324065 (PMC12091782; doi:10.1371/journal.pone.0324065)
Supplement: Attachment — Submitted filename: Comment.docx [file pone.0324065.s001.docx]

Manuscript Number

PONE-D-24-29304

**Abstract:** Validity and reliability of the test should be mentioned.

**Introduction:** The statement of the problem is not mentioned well. What was the need to do this? Explain this problem?

**Materials and Methods**: 1. validity, reliability and source in the tests of maximum strength, throwing the ball forward, backward, right and left.

2. In the anthropometric tests of arm circumference, chest and arm width, refer to the source.

**Discussion: 1.** In general, in the field of strength test, more comprehensive explanations are necessary to complete this section.

2. You have to use more tricks to complete this section
