# Supplementary material for: Anthropometric and strength characteristics of adolescent golfers with low and high handicaps
Source: PLoS One. 2025 May 20;20(5):e0324065. doi: 10.1371/journal.pone.0324065 (PMC12091782; doi:10.1371/journal.pone.0324065)
Supplement: Attachment — Submitted filename: reviwe fail-24-29304.docx [file pone.0324065.s002.docx]

**Review of the Article: "Anthropometric and Strength Characteristics of Adolescent Golfers with Different Handicaps"**

Dear Authors,

To enhance the quality of this article, I kindly suggest the following revisions:

**Title:**

The title should be revised to better reflect the study's objective.

**Suggested Revision:**
"Anthropometric and Strength Characteristics of Adolescent Golfers with Low and High Handicaps."

**Abstract:**

- Please specify the sampling method used and provide a clear explanation of how the sample size was determined.
- The exact p-value should be stated.
- If gender comparisons were made, please indicate this and clarify whether correlations were assessed based on gender.
- The conclusion section should be revised to focus on the implications of the findings rather than restating the results.
- It is advisable to use keywords that are distinct from those already included in the title.
- The abstract appears to be too lengthy. Consider shortening it to a maximum of 250 words.

**Introduction:**

- Ensure that all statements are appropriately cited with references.
- Provide more detailed information about the role of anthropometric characteristics in golfers' performance.
- The literature review largely focuses on the role of strength and power in performance. It is essential to also review studies that explore the significance of anthropometric features in this context.
- While the role of muscular strength and anthropometric characteristics on motor performance is well established, the unique rationale for this research needs further clarification.
- Since anthropometric and physical fitness (strength) assessments serve as entry criteria for most sports and are key to talent identification, please justify the necessity of examining these factors at the adolescent level. Provide educational reasoning for their inclusion in this study.
- Offer a more detailed explanation of how adolescent strength and anthropometric characteristics impact sports performance.
- Numerous studies have explored the importance of anthropometric features and strength in adolescent athletes. Drawing upon relevant studies will strengthen the introduction and provide a more solid foundation.

**Research Method:**

**Participants:**

- Please specify the sampling method and the process for estimating the sample size.
- Considering gender differences among adolescents, how were the groups balanced by gender?
- Were there any other criteria, aside from handicap, used for grouping participants? Please clarify.

**Statistical Analysis:**

- Given the multi-level nature of the research variables, please report the results of multiple regression analysis, if applicable, to determine which variables have the highest predictive power.
- When discussing statistical tests, simply mention the test used and justify its selection. Avoid additional explanations.

**Results:**

- Modify the titles of the tables to better align with the study’s objectives (i.e., “Golfers with Low and High Handicaps”).
- In Table 3, please report the R² value and provide an analysis based on this value.
- A correlation table for the anthropometric characteristics would be beneficial. Please explain why such a table was not included.
- It would be useful to present the results of multiple regression analysis to highlight the predictive power of the strength and anthropometric variables.
- In the methods section, it is stated that the Mann-Whitney U test was used, but this is not mentioned in the results section. Please ensure consistency.

**Discussion:**

- Given the length of the discussion, I suggest you begin with a concise summary of the findings related to anthropometric characteristics and strength analysis. Then, compare these findings with those of previous studies in this area, and conclude with a general summary.
- Given the relationship between limb volume and strength (a key factor in improving performance), I recommend further analysis of the results in terms of their educational applications.
- It might be better to present the correlation section towards the end of the strength discussion.
- Please include practical suggestions based on the findings, particularly from an educational perspective.

**References:**

- The font style in references 2, 5, 12, 26, 29, 39, 42, and 46 appears to be inconsistent. Please correct this formatting issue.
